# The impact of underuse of modern methods of contraception among adolescents with unintended pregnancies in 12 low- and middle-income countries

**DOI:** 10.7189/jogh.09.020429

**Published:** 2019-12

**Authors:** Saverio Bellizzi, Giuseppe Pichierri, Leonardo Menchini, Jessica Barry, Giovanni Sotgiu, Quique Bassat

**Affiliations:** 1 Partnership for Maternal, Newborn & Child Health, Geneva, Switzerland; 2 Kingston Hospital NHS Foundation Trust, Galsworthy Road, Kingston upon Thames, UK; 3 International Labour Organization, Cairo, Egypt; 4 WHO Eastern Mediterranean Regional Office, Cairo, Egypt; 5 Clinical Epidemiology and Medical Statistics Unit, Department of Medical, Surgical and Experimental Sciences, University of Sassari, Italy; 6 ISGlobal, Hospital Clínic - Universitat de Barcelona, Barcelona, Spain; 7 Centro de Investigação em Saúde de Manhiça (CISM), Maputo, Mozambique; 8 ICREA, Barcelona, Spain.; 9 Pediatric Infectious Diseases Unit, Pediatrics Department, Hospital Sant Joan de Déu (University of Barcelona), Barcelona, Spain

## Abstract

**Background:**

In spite of the last decade increase in availability of contraception, around half of the annual 21 million pregnancies notified in low- and middle-income countries in individuals aged 15-19 years are unintended. We sought to explore the contribution of the underuse of modern methods of contraception (MMC) to the annual incidence of unintended pregnancies among adolescent women.

**Methods:**

We used Demographic and Health Survey (DHS) data from 12 low- and middle-income countries. The pooled analysis exploring the risk of unintended pregnancy included 7268 adolescent women with a current unintended pregnancy and 121 894 currently not pregnant 15- to 19-year-old sexually active women who did not desire pregnancy. For each country and the pooled analysis, the odds ratio of unintended pregnancy was calculated in relation to the type of contraception (MMC, Traditional Methods, and No Contraception). Expected unintended pregnancies and population attributable fraction (PAF) of unintended pregnancies attributable to not using MMC were calculated for each country.

**Results:**

The use of traditional methods was associated with a 3.4 (95% confidence interval (CI) = 2.1-4.7) time increased odds of having an undesired pregnancy compared with the use of MMC of contraception while not using any method of contraception was associated with a 4.6 (95% CI = 2.6-6.6) times increased odds. The population attributable fraction (PAF) of not using MMC accounted for 86.8% of the estimated unintended pregnancies (9 464 654 in total in the 12 countries) in the pooled analysis. PAF ranged from 65.8% (1 022 154) for Bangladesh to 95.1% (540 176) for Niger and the estimated number of unintended pregnancies because of the use of traditional methods or non-use of contraception ranged from 18 638 in Namibia to 4 303 872 in India.

**Conclusions:**

Eight million out of 9.5 million unintended pregnancies occurring annually in twelve countries could have been prevented with the optimal use of MMC of contraception. MMC need to be further supported in order to further prevent unintended pregnancies globally.

In the year 2000 several countries committed to decrease maternal mortality implementing various strategies, including improved access to contraception, to achieve the Millennium Development Goal number 5 aimed at improving maternal health [1]. Despite the increase of 61 million adolescent girls with their demand for contraception satisfied with modern methods since 1990 [2], around 23 million adolescent girls annually are at high risk of unintended pregnancies [3]. In 2016, almost half of the 21 million pregnancies estimated in low- and middle-income countries in individuals aged 15-19 years were unintended, ranging from ~ 50% in Africa and Asia up to around 75%in Latin America [3].

Teenage births result in health consequences; children are more likely to be born pre-term, have lower birth weight, and higher neonatal mortality, while mothers experience greater rates of post-partum depression and are less likely to initiate breastfeeding [4,5]. Additionally, adolescent mothers face higher risks of eclampsia, puerperal endometritis, and systemic infections than older women aged 20 to 24 years [6].

Adolescent pregnancy can also have negative economic and social effect on girls and their families, with unmarried pregnant girls likely facing stigma and rejection by parents and peers [7]. With regards to education, school-leaving is often the direct cause of pregnancy: an estimated 5% to 33% of girls who drop out of school in some countries do so because of early pregnancy or marriage [7], which perpetuates the cycle of poverty because of fewer skills and opportunities for employment.

Literature has provided evidence of the association between the uptake of modern contraceptives and socio-economic determinants: living in urban contexts is linked to higher usage when compared to rural residence [8]. Similarly, older, wealthier [9] and higher educated women are more likely to use modern methods of contraception when compared to their counterparts [10]. Adolescents who are not married can face several barriers to access and use contraceptives because sexual activity is only considered acceptable within marriage in many settings [11]. Married adolescents, on the other hand, are often under pressure to have a child soon after marriage and end up pregnant at early ages [11]. Furthermore, partner disapproval of modern contraceptive use, can affect the adoption of modern contraceptives [12].

While considerable research has been conducted to explore use and barriers to use of contraception, no analysis is available on the estimated impact of unmet need for contraception among adolescent girls in low- and middle-income countries. This study estimates the effect of the lack of use of modern methods of contraception (MMC) on unintended pregnancies by assessing the usage of MMC in comparison with traditional methods and with non-use of contraception.

## METHODS

### Data sources

Demographic and Health Surveys (DHS) are large, nationally representative household surveys regularly conducted since the year 1984 in over 90 low- and middle-income countries worldwide [13].

DHS use standardized measurement tools and techniques to ensure comparability across diverse sites and time-periods, and consist of different questionnaires, including a household and a women’s modules [14]. The latter is administered to women of reproductive age (ie, 15–49 years) and includes a contraceptive history calendar for the five years prior to the survey [15].

Since 2010, 36 latest country DHS have incorporated questions regarding contraceptive failure for the current pregnancy, which enables to differentiate from contraception discontinuation followed by early conception.

The answers “later” and “no more” to the DHS question ‘Did you want to have a baby later on or did you not want any (more) children?” were used to construct the variable “wanting to have no more children’ for the currently pregnant girls. Similarly, the answer “no” to the DHS question “Are you currently doing something or using any method to delay or avoid getting pregnant?” and the answers “no more children” and “no children within the next two years” to the question “Would you like to have (a/another) child?” were used to construct the variable ‘not desiring pregnancy and not desiring contraception’ for the currently non-pregnant girls.

Out of the total 36 DHS, 24 surveys were excluded because of the very small sample (fewer than ten) of current unintended pregnancies among girls.

### Variables

The use of contraception was categorized into the following three categories: 1) MMC, including combined oral contraceptives, progestin-only pills, implants, injectable contraceptives, IUDs, male and female condoms, sterilization and LAM [16], 2) traditional methods, including withdrawal and fertility-awareness methods [16], and 3) non-use.

We defined ‘sexually active’ those females engaging in sexual intercourse/s within 30 days of the interview and unintended pregnancy as a pregnancy desired later after occurrence or not desired at all. While status of contraceptive usage for currently non-pregnant women was used at the time of the survey, contraceptive usage for currently pregnant referred to its use immediately prior the information on pregnancy, thus representing contraceptive failure.

Every pregnant woman who discontinued contraception selected one out of six categories primary reason for doing so. ‘Side-effects’ included hormonal contraception-related adverse events; ‘Health concern’ included belief of interference with human physiology; ‘Opposition’ included marital opposition to contraception. ‘Access’ included lack of awareness on how to purchase contraceptives. ‘Inconvenient’ included use-related discomfort. ‘Other’ included less prevalent reasons, such as ‘low sexual frequency and ‘spousal separation.

### Study population and sample size

The risk of unintended pregnancy was computed including currently unintended pregnancies (n = 7268) among 15-19 year-old females and sexually active, currently not pregnant, 15-19 year-old women who did not desire to be pregnant (n = 121 894) at the time of survey. Data were collected in the following 12 countries: Bangladesh 2014, Colombia 2015, Honduras 2011/12, India 2015/16, Liberia 2013, Mozambique 2011, Namibia 2013, Niger 2012, Peru 2012, Sierra Leone 2013, Uganda 2016, and Zambia 2013.

### Statistical analysis

In consideration of the DHS survey design, we accounted for clustering of women’s households by primary sampling units and included country random effect in the analysis.

We tabulated the country specific overall and adolescent female population together with each 15-19 year-old female survey sample size. We also calculated the overall and each country proportion of unintended pregnancies out of all current pregnancies and the proportion of non-desiring children out of all currently non-pregnant girls.

Overall use of modern methods of contraception, traditional methods of contraception and non-use of contraception were calculated for both categories of girl under-study (current unintended pregnancy and currently non-pregnant non-desiring children).

For each country and the pooled analysis, the risk (odds ratio (OR) and adjusted OR (aOR) of unintended pregnancy was assessed in relation to the type of contraception. Logistic regression models included residence (urban or rural), marital status, and wealth (poorest, poor, middle, rich and richest) [17] as covariates. *P*-values less than 0.05 were considered to keep variables in the model.

Adolescent population and adolescent birth rates by country were retrieved from UNICEF State of The World’s Children 2017 Statistical Tables in order to estimate annual expected pregnancies [18]. The estimated number of annual expected pregnancies was calculated as the number of adolescents multiplied by the adolescent birth rate and then by 1.15 to adjust for miscarriages and terminations [19].

Expected unintended pregnancies and population attributable fraction (PAF) of unintended pregnancies attributable to not using MMC were calculated for each country: P(E)(OR–1)/[1+p(E)(OR–1)], where P(E) was the proportion of unintended pregnancies due to non-use of MMC, OR the odds ratio of pregnancy and the use of MMC.

The PAF would provide the proportional reduction in undesired pregnancies if traditional methods of contraception and non-use of contraception at all were replaced by MMC.

The statistical software STATA 13.1 SE was used to perform statistical computations [20].

This analysis did not require additional ethical approval. The analysis relied upon publicly available data set with no identifying data to guarantee participant anonymity. Ethics approval for the survey was obtained by the institutional review board of ORC Macro (Calverton, MD, USA) and country health authorities.

## RESULTS

A total of 30 595 out of 121 894 (25.1%) non-pregnant sexually active women who did not want any future pregnancy were exposed to contraception methods. The analysis evaluating reasons for discontinuing MMC included all adolescent women who discontinued for all reasons but failure. The response rate for fertility preference and intention to use contraception in the non-pregnant women population was >98.0% for all DHS countries.

Of 7268 unintended pregnancies the highest and lowest frequencies were in Peru (113, 71.5%) and Niger (164, 17.8%). Of the 121 894 not pregnant girls who did not desire pregnancy, Mozambique (1367, 50.7%) and Peru 4132 (94.1%) were the less and most represented, respectively (**Table 1**).

**Table 1:** Data on conception obtained from demographic and health surveys in 12 low- and middle-income countries between 2010 and 2016

Country DHS	Total country population (total female adolescent population)	Sample size 15-19 y old	Current unintended pregnancy, n (%)	Currently non-pregnant, adolescent not desiring children, n (%)
Bangladesh 2014	162 952 000 (16 261 000)	1 944	90 (26.3)	1182 (71.1)
Colombia 2015	48 653 000 (4 078 000)	6 604	210 (69.2)	5827 (89.3)
Honduras 2011/12	9 113 000 (995 000)	5 227	132 (38.9)	4220 (85.9)
India 2015/16	1 324 171 000 (125 043 000)	134 751	5927 (29.6)	88 872 (88.1)
Liberia 2013	4 614 000 (521 000)	1 915	109 (60.3)	1171 (66.4)
Mozambique 2011	28 829 000 (3 326 000)	3 065	104 (31.1)	1367 (50.7)
Namibia 2013	2 480 000 (269 000)	1 857	82 (65.2)	1228 (69.9)
Niger 2012	20 673 000 (2 352 000)	7 905	164 (17.8)	5448 (71.3)
Peru 2012	31 774 000 (2 803 000)	4 489	113 (71.5)	4132 (94.1)
Sierra Leone 2013	7 396 000 (852 000)	4 051	118 (46.8)	4259 (80.3)
Uganda 2016	41 488 000 (4 810 000)	2 122	77 (35.5)	1403 (63.9)
Zambia 2013	16 591 000 (1 926 000)	3 686	142 (55.5)	2785 (73.7)

DHS – Demographic and Health Survey, CI – confidence interval

A total of 712 (9.8%) were using MMC, 371 (5.1%) traditional methods of contraception, and 6185 (85.1%) were not using any methods of contraception immediately prior to the current unintended pregnancy. Among non-pregnant and not desiring children adolescent women, 28 401 (23.3%) were using MMC, 3413 (2.8%) traditional methods of contraception, and 90 080 (73.9%) were not using any methods of contraception.

**Figure 1:**
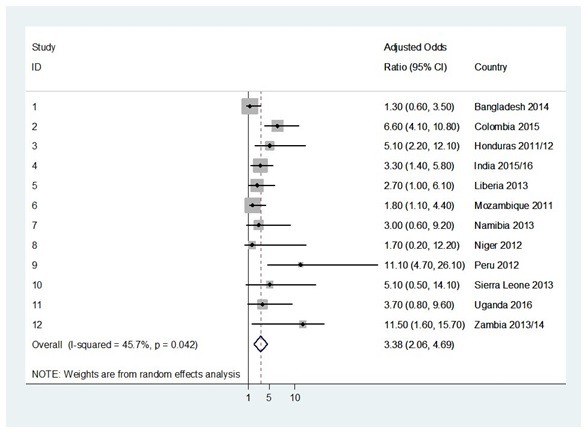
Forest plot showing the adjusted odds ratio of unintended pregnancies in adolescent girls using traditional methods of contraception when compared to adolescent girls using MM of contraception in twelve low- and middle- income countries in the survey between 2010 and 2016.

**Figure 2:**
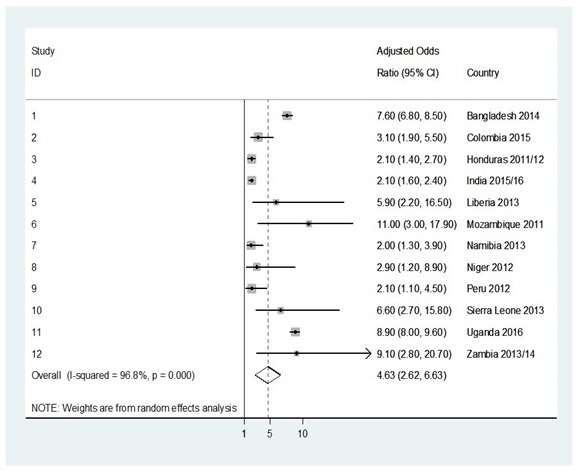
Forest plot showing the adjusted odds ratio of unintended pregnancies in adolescent girls non-using any methods of contraception when compared to adolescent girls using MM of contraception in twelve low- and middle- income countries in the survey between 2010 and 2016.

In the pooled analysis, use of traditional methods or non-use of any methods was associated with an increased risk of having an unintended pregnancy (OR = 3.4, 95% confidence interval (CI) = 2.1-4.7; OR = 4.6, 95% CI = 2.6-6.6, respectively) (**Figure 1** and **Figure 2**).

Country-specific OR of unintended pregnancy associated with using traditional methods ranged from 1.3 (95% CI = 0.6-3.5) for Bangladesh to 11.1 (95% CI = 4.7-26.1) for Peru, with statistically significant results for 7/12 (58.3%) countries. Country-specific OR of unintended pregnancy associated with the non-use of any contraception methods ranged from 2.0 (95% CI = 1.3-3.9) for Namibia to 11.0 (95% CI = 3.0-17.9) for Mozambique, with statistically significant findings for all 12 (100.0%) surveyed countries.

The PAF of not using MMC accounted for 86.8% (8 220 605) of the estimated unintended pregnancies (projected to be 9 464 654) in the pooled analysis. The PAF ranged from 65.8% (1 022 154) for Bangladesh to 95.1% (540 176) for Niger and the estimated number of unintended pregnancies because of the use of traditional methods or non-use of contraception ranged from 18 638 in Namibia to 4 303 872 in India (**Table 2**).

**Table 2:** Population Attributable Fraction of annual unintended pregnancies for not using modern methods of contraception among adolescent women in 12 low- and middle-income countries between 2010 and 2016

Country, survey, years	Annual unintended pregnancies	Population attributable fraction (95% CI)	Number of annual unintended pregnancies due to non-use of MM
Pooled	9 464 654	86.8 (85.4-88.3)	8 220 605 (8 107 371-8 411 098)
Bangladesh 2014	1 554 642	65.8 (53.2-72.5)	974 760 (814 632-1 145 771)
Colombia 2015	397 794	82.2 (80.0-84.4)	326 987 (318 235-335 738)
Honduras 2011/12	113 281	89.8 (88.6-91.0)	101 726 (100 367-103 085)
India 2015/16	4 608 179	93.4 (92.8-94.1)	4 303 872 (4 201 112-4 373 341)
Liberia 2013	87 991	88.6 (87.3-89.9)	77 960 (76 816-79 104)
Mozambique 2011	634 647	88.7 (87.2-89.6)	562 932 (553 412-568 644)
Namibia 2013	24 174	77.1 (75.0-79.9)	18 638 (18 130-19 315)
Niger 2012	568 008	95.1 (94.5-95.6)	540 176 (536 767-543 016)
Peru 2012	219 195	92.7 (92.0-93.5)	203 194 (201 659-204 947)
Sierra Leone 2013	128 354	82.4 (81.2-84.4)	105 764 (104 223-108 331)
Uganda 2016	798 560	90.9 (89.5-92.8)	712 263 (700 409-727 529)
Zambia 2013	329 832	89.5 (88.3-90.1)	295 200 (291 242-297 179)

CI – confidence interval, MM – modern methods

## DISCUSSION

The study carried out in twelve low- and middle-income countries showed that ~ 9/10 unintended pregnancies would have been averted if MMC were utilized. The use of traditional methods and not using any methods of contraception significantly increased the odds of an unintended pregnancy, respectively.

Around 85% of adolescent women with a current unintended pregnancy and 75% of non-pregnant, not desiring children adolescent women were not using any contraception, which is the classical “unmet need” [21]. On the other hand, 5% and 3% of the two populations under study were making use of traditional methods, which entail a very high risk of unintended pregnancy as well as high STI/HIV exposure [22].

The surveyed countries record ~ 10 million unintended adolescent pregnancies annually, representing around 40% of the unintended pregnancies among adolescent women worldwide. In all countries adolescent girls had significant risk of unintended pregnancy associated with the non-use of contraception, whereas more than 50% had significant risk of unintended pregnancy associated with the traditional methods of contraception.

The high proportion (>¼) of women in Bangladesh and Namibia who became pregnant despite using MMC needs to be carefully evaluated. As emphasized by a 2014 policy brief, in Bangladesh there has been an important decline of the use of long-acting modern methods of contraception in the last decades; the increased adoption of short-acting modern methods of contraception and their incorrect use together with high rates of contraception discontinuation is slowing down the progress made in the past 20 years [23].

Furthermore, One out of four adolescent women with an unintended pregnancy discontinued MMC because of adverse events, confirming their role for discontinuation of drugs, injectables, and IUDs in low-income settings [24]. Discontinuation is a specifically important issue for adolescent due to the fact that they tend to have a more unpredictable and irregular sexual activity, more limited access than older individuals to family planning, and usually less knowledge about effective use of contraceptive methods [25]. More attention should be paid to social and health care factors, such as lack of access/availability and partner disapproval.

DHS are often the only nationally-representative source of reproductive health information in low- and middle-income countries and are generally considered of high accuracy [26]. However, DHS findings were compared across ten countries in different periods after 2005. However, standardized procedures and questionnaires were implemented minimizing the methodological variability [26].

More than 95% of the eligible women took part in all surveys under study, with Honduras having the lowest participation rates (95%) and Niger the highest (98%).

Post-event rationalization bias [27] on the intention status of pregnancies should have been minimized since with the analysis was focused on current pregnancies. Scientists have also argued on the validity of the meaning of “unintendedness”, following the contradiction on happiness pregnancy is diagnosed [28,29].

Further, the high heterogeneity within the category of unintended pregnancy should be carefully considered, with unwanted and mistimed pregnancies representing different life-choice considerations [30].

Moreover, recall bias on the contraception use prior to the current pregnancy could potentially affect the results [31]: women who experienced an unintended pregnancy might be more likely to recall and report their sexual behaviours compared with those who did not have any unintended pregnancy. Regular and frequent contraceptive use such as daily pill use, regular injections, or consistent use of an IUD could be expected to be more accurately recalled than coitally dependent methods such as condom use and withdrawal, which are practiced more sporadically and infrequently [32]. Underreporting of certain methods such as condoms has been in fact documented in different studies, especially in West Africa [33].

As showed by several reviews [34,35], adolescent face barriers in obtaining and using modern contraception in low- and middle-income settings (eg, drug shortages) [36,37]; furthermore, when they are available, health care workers and/or local policies cannot provide family planning services to adolescents or those under a certain age [36]. Stigma on contraception can prevent their use by adolescents not in stable relationship [38].

Fear and concerns related to misconception on early and long-term adverse events can lead young women to consider more acceptable withdrawal and other traditional methods [39].

Moreover, poor understanding of how contraceptives methods works, and consistent use of contraception has been shown to be problematic among adolescents [40]. Unintended pregnancies often occur during periods when women engage in contraceptive switching, often to less effective methods, or when abandon contraception [41,42]. The rate of switching to no method is key: 85% can become pregnant in the first year after stopping [43]. A DHS report showed that adolescent women are at high risk of discontinuation when compared to older women [44]. In this regard, consistent male condom’s use, which is the most commonly used method by adolescents due to accessibility and price [36], tends to decrease over time within stable partnerships [45].

A recent Cochrane review [46] showed that promoting the use of contraceptive measures did not reduce the risk of unintended pregnancies; however, multiple interventions (ie, educational, skill building and contraception promotion) can reduce the rate of unintended pregnancies in adolescents [47,48].

A recent study [49] underscored that 90% of adolescent women used short-acting methods (eg, condoms, pills, injectables) and that an increase in the use of long-acting reversible methods (eg, IUDs and implants) would decrease the cost.

In conclusion, our study estimated that 8 million unintended pregnancies in 12 low- and middle-income countries could have been prevented annually if all adolescent women who did not desire pregnancy had used modern methods of contraception. It is fundamental to understand how under use of MMC translates into a disease and mortality burden. Systematic efforts are needed to address issues like access to contraception, fear of adverse events, opposition to use and underestimation of the risk of pregnancy.

Combined approach with multi-sectoral approach including health facility (antenatal, immediate post-natal and post-abortive care visits), school (sexual education), national policies and laws, as well as the use of media describing accurate and context-tailored information, are recommended to advance towards the Sustainable Development Goal 3 [50]. This is also in line with the Universal Health Coverage Agenda, which specifically calls for the inclusion of core sexual and reproductive health in health services by providing a level of financial protection sufficient to insulate adolescent against economic hardship [51].

## References

[1] World Health Organization. (WHO). MDG 5: improve maternal health. 2015. Available: https://www.who.int/topics/millennium_development_goals/maternal_health/en/. Accessed: 26 June 2019.

[2] Azzopardi PS Hearps SJC Francis KL Kennedy EC Mokdad AH Kassebaum NJ Progress in adolescent health and wellbeing: tracking 12 headline indicators for 195 countries and territories, 1990-2016. Lancet. 2019;393:1101-18. 10.1016/S0140-6736(18)32427-9 30876706 PMC6429986

[3] Darroch JE, Woog V, Bankole A, Ashford LS. Adding it up: costs and benefits of meeting contraceptive needs of adolescents. New York (NY): Guttmacher Institute. 2016.

[4] Chen XK Wen SW Fleming N Demissie K Rhoads GG Walker M Teenage pregnancy and adverse birth outcomes: a large population based retrospective cohort study. Int J Epidemiol. 2007;36:368-73. 10.1093/ije/dyl284 17213208

[5] Kingston D Heaman M Fell D Chalmers B Comparison of adolescent, young adult, and adult women’s maternity experiences and practices. Pediatrics. 2012;129:e1228-37. 10.1542/peds.2011-1447 22529278

[6] Ganchimeg T Ota E Morisaki N Laopaiboon P Zhang J Yamdamsuren B Pregnancy and childbirth outcomes among adolescent mothers: a World Health Organization multicountry study. BJOG. 2014;121 Suppl 1:40-8. 10.1111/1471-0528.12630 24641534

[7] World Bank. Economic impacts of child marriage: Global Synthesis report. Washington, DC: World Bank; 2017.

[8] Audu B Yahya S Geidam A Abdussalam H Takai I Kyari O Polygamy and the use of contraceptive. Int J Gynaecol Obstet. 2008;101:88-92. 10.1016/j.ijgo.2007.09.036 18082747

[9] Gakidou E Vayena E Use of modern contraception by the poor is falling behind. PLoS Med. 2007;4:e31. 10.1371/journal.pmed.0040031 17284155 PMC1796626

[10] Nagase T Kunii O Wakai S Khaleel A Obstacles to modern contraceptive use among married women in Southern Urban Maldives. Contraception. 2003;68:125-34. 10.1016/S0010-7824(03)00113-6 12954525

[11] de Vargas Nunes Coll C Ewerling F Hellwig F de Barros AJD Contraception in adolescence: the influence of parity and marital status on contraceptive use in 73 low-and middle-income countries. Reprod Health. 2019;16:21. 10.1186/s12978-019-0686-9 30791914 PMC6383262

[12] Alio AP Daley EM Nana PN Duan J Salihu HM Intimate partner violence and contraceptive use among women in Sub-Saharan Africa. Int J Gynaecol Obstet. 2009;107:35-8. 10.1016/j.ijgo.2009.05.002 19481751

[13] Macro O. Demographic and Health Surveys Methodology Interviewer’s Manual, 2012 Calverton, MD ORC Macro. Available: https://dhsprogram.com/pubs/pdf/DHSG1/Guide_to_DHS_Statistics_29Oct2012_DHSG1.pdf. Accessed: 26 June 2019.

[14] Pullum TW. An Assessment of the Quality of Data on Health and Nutrition in the DHS Survey, 1993-2003, 2008 Calverton, MD Macro International Inc. Available: https://dhsprogram.com/pubs/pdf/MR6/MR6.pdf. Accessed: 26 June 2019.

[15] Marriott BM Campbell L Hirsch E Wilson D Preliminary data from demographic and health surveys on infant feeding in 20 developing countries. J Nutr. 2007;137:518S-23S. 10.1093/jn/137.2.518S 17237339

[16] World Health Organization. Family planning/Contraception. Available: http://www.who.int/news-room/fact-sheets/detail/family-planning-contraception. Accessed: 26 June 2019.

[17] Rustein SO, Johnson K. The DHS Wealth Index. Calverton: ORC Macro; 2004. Available: https://dhsprogram.com/pubs/pdf/CR6/CR6.pdf. Accessed: 26 June 2019.

[18] UNICEF. State of The World’s Children 2017 Statistical Tables. Available: https://data.unicef.org/resources/state-worlds-children-2017-statistical-tables. Accessed: 26 June 2019.

[19] Garcia-Enguidanos A Calle ME Valero J Luna S Dominguez-Rojas V Risk factors in miscarriage: a review. Eur J Obstet Gynecol Reprod Biol. 2002;102:111-9. 10.1016/S0301-2115(01)00613-3 11950476

[20] Stata Corp. Statistical Software: Release 13SE [Computer Program]. College Station, TX: Stata Corp.; 2013.

[21] World Health Organization. Unmet need for family planning. Available: http://www.who.int/reproductivehealth/topics/familyplanning/unmetneedfp/en. Accessed: 26 June 2019.

[22] Bellizzi S Sobel HL Obara H Temmerman M Underuse of modern methods of contraception: underlying caused and consequent undesired pregnancies in 35 low- and middle-income countries. Hum Reprod. 2015;30:973-86. 10.1093/humrep/deu348 25650409

[23] Reduce contraception discontinuation in Bangladesh by improving counseling on side effects. STEP UP. Strengthening evidence for programming on Unintended Pregnancy. POP Council. 2014. Available: https://www.popcouncil.org/uploads/pdfs/2014STEPUP_ContraceptionDiscontinuation.pdf. Accessed: 26 June 2019.

[24] Mumah JN Machiyama K Mutua M Kabiru CW Cleland J Contraceptive adoption, discontinuation, and switching among postpartum women in Nairobi’s Urban Slums. Stud Fam Plann. 2015;46:369-86. 10.1111/j.1728-4465.2015.00038.x 26643488 PMC5064637

[25] Blanc AK Tsui AO Croft TN Trevitt JL Patterns and trends in adolescents’ contraceptive use and discontinuation in developing countries and comparisons with adult women. Int Perspect Sex Reprod Health. 2009;35:63-71. 10.1363/3506309 19620090

[26] Johnson K, Grant M, Khan S, Moore Z, Armstrong A, Sa Z. Fieldwork-Related Factors and Data Quality in the Demographic and Health Surveys Program, 2009 Calverton, MD Macro International. Available: ttps://www.dhsprogram.com/publications/publication-as19-analytical-studies.cfm. Accessed: 26 June 2019.

[27] Curtis S Evens E Sambisa W Contraceptive discontinuation and unintended pregnancy: an imperfect relationship. Int Perspect Sex Reprod Health. 2011;37:58-66. 10.1363/3705811 21757420 PMC3912075

[28] Santelli J Rochat R Hatfield-Timajchy K Gilbert BC Curtis K Cabral R The measurement and meaning of unintended pregnancy. Perspect Sex Reprod Health. 2003;35:94-101. 10.1363/3509403 12729139

[29] Trussell J Vaughan B Stanford J Are all contraceptive failures unintended pregnancies? Evidence from the 1995 National Survey of Family Growth. Fam Plann Perspect. 1999;31:246-7, 260. 10.2307/2991573 10723650

[30] Luker KC A reminder that human behaviour frequently refuses to conform to models created by researchers. Fam Plann Perspect. 1999;31:248-9. 10.2307/2991574 10723651

[31] Boerma JT Sommerfelt AE Demographic and health survey (DHS): contributions and limitations. World Health Stat Q. 1993;46:222-6. 8017081

[32] Callahan RL Becke S The reliability of calendar data for reporting contraceptive use: evidence from rural Bangladesh. Stud Fam Plann. 201243:213-22. 10.1111/j.1728-4465.2012.00319.x 23185864 PMC3628694

[33] Calverton MD. USA: 2015. Contraceptive use and perinatal mortality in the DHS: an assessment of the quality and consistency of calendars and histories. Available: https://www.dhsprogram.com/pubs/pdf/MR17/MR17.pdf. Accessed: 26 June 2019.

[34] Williamson LM Parkes A Wight D Petticrew M Hart GJ Limits to modern contraceptive use among young women in developing countries: a systematic review of qualitative research. Reprod Health. 2009;6:3. 10.1186/1742-4755-6-3 19228420 PMC2652437

[35] Marston C King E Factors that shape young people’s sexual behaviour: a systematic review. Lancet. 2006;368:1581-6. 10.1016/S0140-6736(06)69662-1 17084758

[36] Bankole A Malarcher S Removing barriers to adolescents’access to contraceptive information and services. Stud Fam Plann. 2010;41:117-24. 10.1111/j.1728-4465.2010.00232.x 21466111

[37] Nalwadda G Mirembe F Tumwesigye NM Byamugisha J Faxelid E Contraints and prospects for contraceptive service provision to young people in Uganda: Providers’ perspectives. BMC Health Serv Res. 2011;11:220. 10.1186/1472-6963-11-220 21923927 PMC3181204

[38] Castaneda X Brindis C Camey IC Nebulous margins: sexuality and social constructions of risks in rural areas of Central Mexico. Cult Health Sex. 2001;3:203-19. 10.1080/136910501750153021

[39] Wood K Jewkes R Blood blockages and scolding nurses: barriers to adolescent contraceptive use in South Africa. Reprod Health Matters. 2006;14:109-18. 10.1016/S0968-8080(06)27231-8 16713885

[40] Chandra-Mouli V McCarraher DR Phillips SJ Williamson NE Hainsworth G Contraception for adolescents in low and middle income countries: needs, barriers, and access. Reprod Health. 2014;11:1. 10.1186/1742-4755-11-1 24383405 PMC3882494

[41] Hossain MB Analysing the relationship between family planning worker’s contact and contraceptive switching in rural Bangladesh using multilevel modelling. J Biosoc Sci. 2005;37:529-54. 10.1017/S0021932004007096 16174345

[42] Vaughan B Trussel J Kost K Singh S Jones R Discontinuation and resumption of contraceptive use: results from the 2002 National Survey of Family Growth. Contraception. 2008;78:271-83. 10.1016/j.contraception.2008.05.007 18847574 PMC2800035

[43] Trussell J Contraceptive failure in the United States. Contraception. 2011;83:397-404. 10.1016/j.contraception.2011.01.021 21477680 PMC3638209

[44] Blanc AK Tsui AO Croft TN Trevitt JL Patterns and trends in adolescents’ contraceptive use and discontinuation in developing countries and comparisons with adult women. Int Perspect Sex Reprod Health. 2009;35:63-71. 10.1363/3506309 19620090

[45] Biddlecom AE, Hessburg L, Singh S, Bankole A, Darabi L. Protecting the next generation in Sub-Saharan Africa: learning from adolescents to prevent HIV and unintended pregnancy. New York: Guttmacher Institute; 2017. Available: https://www.guttmacher.org/report/protecting-next-generation-sub-saharan-africa. Accessed: 26 June 2019.

[46] Oringanje C Meremikwu MMC Eko H Esu E Meremikwu A Ehiri JE Interventions for preventing unintended pregnancies among adolescents. Cochrane Database Syst Rev. 2016;2:CD005215. 10.1002/14651858.CD005215.pub3 26839116 PMC8730506

[47] Kirby D The impact of schools and school programs upon adolescent sexual behaviour. J Sex Res. 2002;39:27-33. 10.1080/00224490209552116 12476253

[48] Manlove J, Terry-Humen E, Papillo RA, Franzentta K, Williams S, Ryan S. Preventing teenage pregnancy, childbearing and STDs [What the research shows]. Child Trends Research Brief. Available: https://eric.ed.gov/?id=ED465150. Accessed: 26 June 2019.

[49] Biddlecom A, Riley T, Darroch JE, Sully E. Future scenarios of Adolescents Contraceptive Use, Cost and Impact in Developing Regions. New York: Guttmacher Institute; 2018. Available: https://www.guttmacher.org/report/adolescent-contraceptive-use-in-developing-regions. Accessed: 26 June 2019.

[50] Sustainable Development Goal 3. Ensure health lives and promote well-being for all at all ages. Available: https://sustainabledevelopment.un.org/sdg3. Accessed: 26 June 2019.

[51] Waddington C Sambo C Financing health care for adolescents: a necessary part of universal health coverage. Bull World Health Organ. 2015;93:57-9. 10.2471/BLT.14.139741 25558109 PMC4271683

